# Quadratic Age Patterns in Self-Perceptions of Aging: The Moderating Role of Educational Attainment

**DOI:** 10.1093/geroni/igaf122.113

**Published:** 2025-12-31

**Authors:** Charles Johnson, Yanjun Dong, Wenxing Wei, Zexi Zhou, Kun Wang

**Affiliations:** The University of Alabama at Birmingham, Birmingham, Alabama, United States; SUNY at Albany, Albany, New York, United States; Case Western Reserve University, Cleveland, Ohio, United States; The University of Texas at Austin, Austin, Texas, United States; University of Alabama at Birmingham, Birmingham, Alabama, United States

## Abstract

Higher education often serves as a protective factor for health and psychological well-being among older adults. Beyond providing access to socioeconomic resources, education fosters problem-solving skills, knowledge, and a sense of agency, enabling individuals to manage aging-related challenges effectively. Consequently, older adults with higher educational attainment may hold higher expectations for aging than their less-educated peers. However, as they age, functional declines and physical limitations beyond their control may emerge. According to Discrepancy Theory, dissatisfaction arises when a gap exists between expectations and reality, suggesting that highly educated older adults may experience more pronounced changes in self-perceptions of aging (SPA). Yet, this relationship remains unexplored. This study 1) examined how positive and negative SPA vary across different ages, and 2) investigated how educational attainment moderates these associations. Data from the Health and Retirement Study (HRS) included adults aged 50+ who completed the Leave-Behind Questionnaire in 2008/2010 (baseline n = 14,538) and were followed through 2012/2014 and 2016/2018. Mixed-effects models with quadratic age terms and interactions between age and education were used. Results showed quadratic trajectories for SPA: positive SPA followed an inverted U-shape, peaking around age 74, while negative SPA exhibited a U-shape, increasing with age. Educational attainment significantly moderated these patterns (Positive SPA: B = -0.01, p < .001; Negative SPA: B = 0.01, p < .001), with higher educational attainment linked to sharper changes in SPA over the lifespan. Tailored interventions should address the unique psychological needs of highly educated older adults to support positive aging perceptions.

